# Ancient Urban Ecology Reconstructed from Archaeozoological Remains of Small Mammals in the Near East

**DOI:** 10.1371/journal.pone.0091795

**Published:** 2014-03-12

**Authors:** Lior Weissbrod, Dan Malkinson, Thomas Cucchi, Yuval Gadot, Israel Finkelstein, Guy Bar-Oz

**Affiliations:** 1 Zinman Institute of Archaeology, University of Haifa, Haifa, Israel; 2 Department of Geography, University of Haifa, Haifa, Israel; 3 The Golan Research Institute, University of Haifa, Katzrin, Israel; 4 UMR 7209 “Archéozoologie, Archéobotanique: Sociétés, Pratiques et Environnements,” Centre National de la Recherche Scientifique/ Muséum National d’Histoire Naturelle, Paris, France; 5 Department of Archaeology, University of Aberdeen, Aberdeen, United Kingdom; 6 The Jacob M. Alkow Department of Archaeology and Ancient Near Eastern Civilizations, Tel Aviv University, Tel Aviv, Israel; University of Florence, Italy

## Abstract

Modern rapidly expanding cities generate intricate patterns of species diversity owing to immense complexity in urban spatial structure and current growth trajectories. We propose to identify and uncouple the drivers that give rise to these patterns by looking at the effect of urbanism on species diversity over a previously unexplored long temporal frame that covers early developments in urbanism. To provide this historical perspective we analyzed archaeozoological remains of small mammals from ancient urban and rural sites in the Near East from the 2nd to the 1st millennium BCE, and compared them to observations from modern urban areas. Our data show that ancient urban assemblages consistently comprised two main taxa (*Mus musculus domesticus* and *Crocidura* sp.), whereas assemblages of contemporaneous rural sites were significantly richer. Low species diversity also characterizes high-density core areas of modern cities, suggesting that similar ecological drivers have continued to operate in urban areas despite the vast growth in their size and population densities, as well as in the complexity of their technologies and social organization. Research in urban ecology has tended to emphasize the relatively high species diversity observed in low-density areas located on the outskirts of cities, where open and vegetated patches are abundant. The fact that over several millennia urban evolution did not significantly alter species diversity suggests that low diversity is an attribute of densely-populated settlements. The possibility that high diversity in peripheral urban areas arose only recently as a short-term phenomenon in urban ecology merits further research based on long-term data.

## Introduction

Interaction between the patterns of urban growth and development and the ecology within modern cities is rarely addressed in current research. This is surprising given that these two contemporary and prevalent concerns, namely development and ecological dynamics, and consequently conservation of species diversity, are intimately connected. The paucity of research may reflect in part the difficulties encountered in studying modern urban settings because of their enormously complex structural and spatial characteristics. Research in the late 1980s by Dickman [1] showing unexpectedly high species diversity within the city of Oxford, England heralded an escalation in research on urban ecology [2]–[5]. This finding had important implications for conservation and human wellbeing in that it indicated that urban species diversity is related to habitat quality [6] (e.g., increase in green spaces and pollution control). Yet, from the point of view of urban planning, spatial structure, and growth patterns, the phenomenon of high species diversity is closely related to expansion of low-density residential areas in the urban fringe [7]. The desirability of such low-density urban sprawl, which incorporates substantial open spaces at the urban fringe, has been a subject of intense debate since the middle of the 20^th^ century [8].

We present a new perspective on these crucial issues by reconstructing the diversity of species of ancient urban settlements. We use data from a wide range of archaeological samples taken from numerous urban and rural sites in the Near East, an early center of urbanization. We examine species composition and diversity in the ancient sites and compare our data with published data from modern urban settlements. Understanding how ecological conditions in urban areas have responded to long-term trajectories of urban growth may help to clarify the role that current urban development plays in matters of ecology and conservation. Modern urban expansion over wide areas results in massive habitat transformation, which is coupled with reduced species diversity and leads to biological homogenization globally [9]. We seek to determine whether the relatively high species diversity that is associated with modern low-density urban development is a recent threshold phenomenon or merely a deviation from a pattern that has been stable for a long time. Answers to these questions have major implications for current concerns regarding conservation and urban planning and for our ability to predict future changes in the nature of densely settled human environments.

Lessons learned from thousands of years of urbanization processes in different parts of the world have been largely overlooked by researchers outside of archaeology [10]. Over these time spans urban environments have been extensively transformed by vast growth in size and density [11]. Such intensification would have been predicated on increasing complexity of economic and social systems and on large-scale technological advancements [11]–[15]. It has been argued that as the size of settlements increases over long time spans so does the population density of human settlements, and that consequently modern low-density urban sprawl is a deviation from a prolonged stable pattern and is unsustainable in the long term [11]. Recent archaeological research in what is considered the earliest example of incipient urbanization at the site of Tel Brak in northern Syria demonstrated a correlative increase in settlement size and physical density along a timeline of 800 years between 4200 and 3400 cal BCE [16].

**Figure 1:**
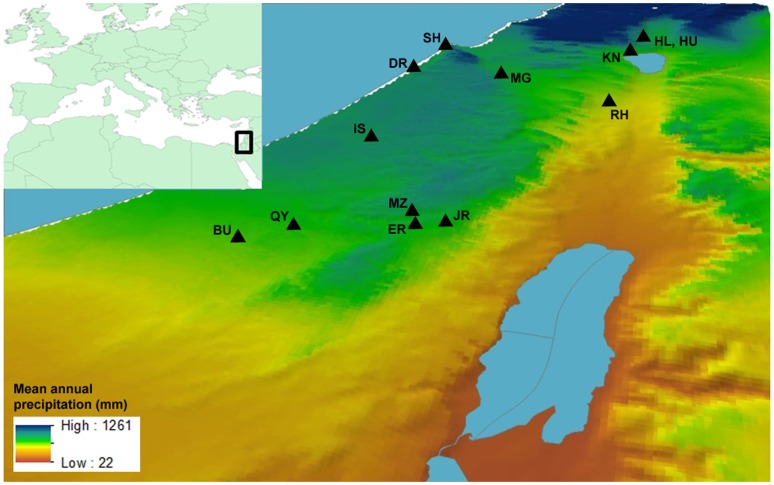
Location map of the study sites in relation to climate and topography.

**Figure 2:**
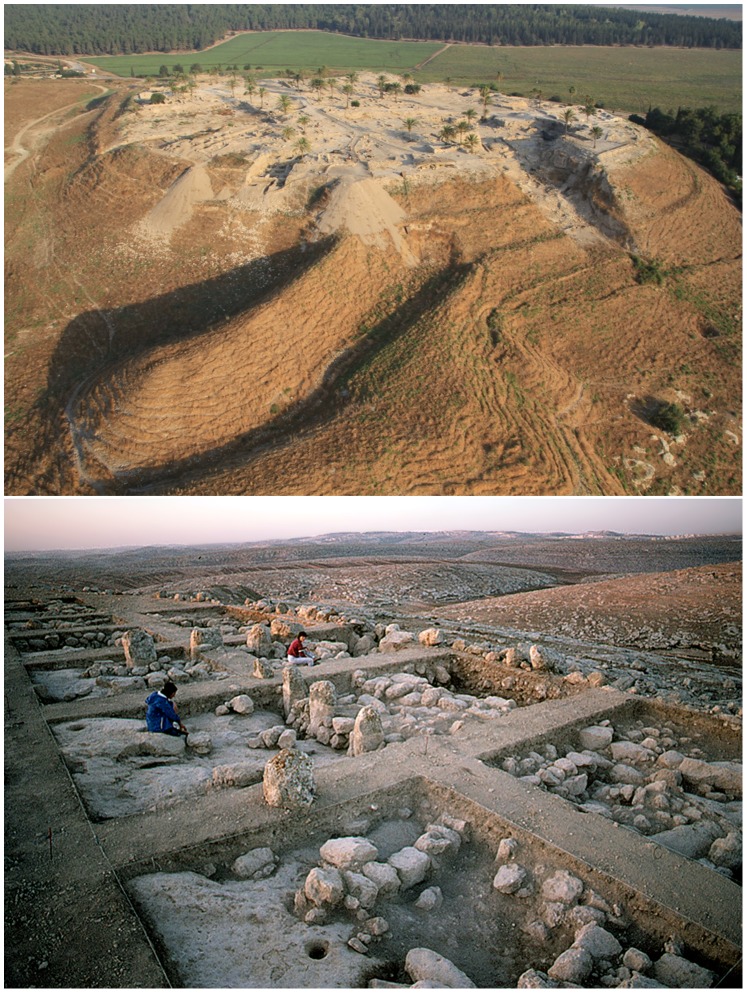
Contrast between a large-scale mound site and a single-period small-scale settlement. Tel Megiddo has a history of thousands of years of dense urban occupation (above; photographed by Skyview and the Megiddo Expedition) whereas Khirbet ed-Dawwara is a single-period small-scale settlement with shallow accumulation above the natural hill topography (below; photographed by I. Finkelstein). The original rocky surface of Khirbet ed-Dawara can be glimpsed in the excavation area only slightly below the present-day surface and in the surrounding hilly landscape. This site is a fortified rural settlement situated in the Jerusalem area and dated to the Iron Age IIA period at the beginning of the 1st millennium BCE.

The sites of the present study are concentrated in the southwestern part of the Near East (Figure 1). The physical consequences of ancient Near Eastern urbanization included large-scale accumulation of mounds and radical transformation of the original substrate over time. In contrast to rural, low-density sites, which are characterized by shallow sediment accumulation, the formation of mounds testifies to continuous and dense long-term settlement [17]–[18] (Figure 2). All of the study sites are situated within or adjacent to the Mediterranean climate and vegetation zone of Israel (mean annual precipitation > 400 mm) and represent the three major geographic formations of the region: coastal plain, central highland, and inland plain. Urbanization in this part of the Near East began later and developed initially on a more modest scale than in other early Near Eastern centers of urban development, including northern Syria and southern Iraq, in the 5^th^–4^th^ millennia BCE [19]–[21]. We focus, however, on the later period of well-established and fairly stable urbanism in the latter half of the 2^nd^ and first half of the 1^st^ millennium BCE, which encompass the Late Bronze Age and the Iron Age. During that period there was considerable variation in the size of urban settlements owing to differences in geographic location or temporal developments [22]–[24].

Our data comprise the taxonomic group of small mammals, including rodents and insectivores <0.5 kg in body mass. We focused on this particular group because the remains are relatively ubiquitous in archaeological deposits of sites in the study area in comparison to other small vertebrate groups [25]. Also, unlike the remains of other animals such as livestock or pets, which were intentionally introduced to settlements by humans, small mammals would have been human commensals and their remains probably accumulated independently. We collected archaeozoological skeletal remains of small mammals in different excavation areas, contexts, or temporal phases of multiple archaeological sites (Table S1). Eleven different assemblages were collected from different chronological phases in six urban sites and six additional assemblages from three rural ones. To gain a detailed understanding of the variation among ancient urban settlements we included assemblages from sites of widely varying sizes as well as four additional unclassified assemblages from contexts of abandoned or off-mound urban sites (Table S1). The total number of sites included in the study is thirteen.

An important limitation of archaeozoological data is that accurate ecological interpretation depends on an understanding of the mode of accumulation of the remains [26]. Our interest is in the remains of animals that lived and died within ancient settlements; we refer to these remains as the occupational community of the settlement. Other potential taphonomic modes may be associated more closely with periods of settlement abandonment. These include predators that deposited the remains of their prey in abandoned structures [27]–[29] and tunneling species that may have burrowed into archaeological sediments and died in the burrows, creating temporally intrusive assemblages [30]. Remains originating from contexts of abandoned sites referred to here as abandonment communities are of interest because, when taken together, occupational and abandonment communities provide a more complete picture of the ecology of sites with long histories of human occupation than that obtained by either type of community alone.

We look at taxonomic lists per site as a broad approach to analysis of the ecology of ancient urban mounds. We also zoom in to reconstruct detailed taxonomic frequencies in the material associated with discrete phases of occupation of the ancient settlements. Combining these two levels of analysis gives us a more realistic assessment of observed patterns in the data and a basis for more reliable comparison of ancient and modern urban ecologies. To assess the influence of occupation versus abandonment on accumulation of the remains, we use proxy assemblages from different archaeological and modern sources to represent the different types of communities from contexts of abandoned sites (see Table S1 and Materials and Methods).

## Results

We analyzed the archaeozoological data based on counts of skeletal specimens identified to taxon, expressed as the number of identified specimens (NISP; Table 1). The total NISP across 21 assemblages from 13 sites is 1,619 and the number of taxa is ten. We assessed potential effects on variation in the data including sample size and inter-site variation in background taxonomic richness. A sample size effect is indicated by a correlation between the NISPs and the number of taxa in the assemblages (*r* = 0.681, *P* = 0.01, n = 13). We examined this relationship through an analysis of nestedness, which tests whether taxonomically poorer assemblages represent random subsets of assemblages that are richer in terms of the incidence of taxa. The analysis conducted through the NeD web system [31] uses three different indices of nestedness including the Brualdi and Sanderson discrepancy index (BR) and a fixed-fixed null model algorithm as recommended by Ulrich and Gotelli [32] (see also [33]). The results of the three indices indicate that we should reject the null hypothesis of a nested pattern (NODF = 80.481, Z = 0.328, *P*>0.05; NODF_row = 84.907, Z = 0.38, *P*>0.05; NODF_col = 77.927, Z = 0.035, *P*>0.05; T = 10.6, Z = −1.361, *P*>0.05; BR = 6.0, Z = −1.315, *P*>0.05) suggesting that the differences in numbers of taxa among assemblages are related to more substantial factors than sampling effects.

**Table 1:** Taxonomic incidence and sample sizes across the study sites.

Settlement type	Site (Abb.*)	*Acomys*	*Apodemus*	*Cricetulus*	*Crocidura*	*Erinaceus*	*Gerbillus*	*Meriones*	*Microtus*	*Mus*	*Spalax*	NISP	#Taxa
Rural	IS	+	−	−	+	−	−	+	+	+	+	82	6
	QY	+	−	+	+	+	+	+	+	+	+	261	9
	ER	+	+	+	+	−	+	+	+	+	+	296	9
Urban	HU	−	−	−	+	−	−	+	+	+	−	49	4
	KN	−	−	−	+	−	−	+	−	+	−	57	4
	SH	−	−	−	+	−	−	+	−	+	−	79	3
	DR	−	−	−	+	−	−	+	+	+	−	260	4
	MG	−	−	−	+	−	+	+	+	+	+	220	6
	RH	−	−	−	+	−	−	+	−	+	+	157	4
Unclass−ified	Hl	−	−	−	−	+	−	+	+	+	+	50	5
	MZ	+	−	−	+	−	−	+	−	+	−	25	4
	JR	+	−	−	+	−	−	−	−	+	−	42	3
	BU	+	−	−	+	−	−	+	+	+	−	41	5
Total												1619	

*For site names see Table S1.

**Figure 3:**
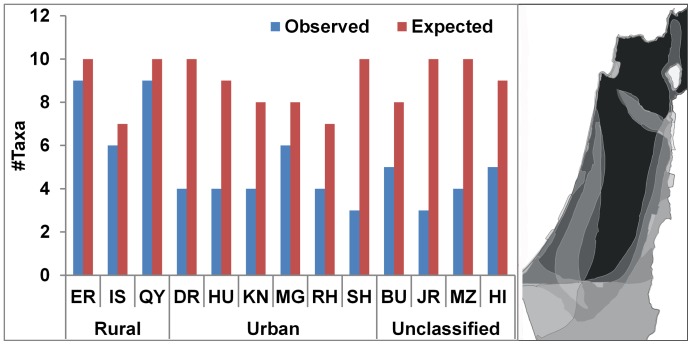
Observed and expected taxonomic richness (number of taxa) in urban and rural sites. Expected richness is based on modern distributions of species modeled with GIS. Distribution map of taxonomic richness for the taxa included in this study, based on maps in Mendelssohn and Yom-Tov [62] is shown on right. Darker shades in the map indicate higher numbers of taxa.

The differences observed between urban and rural sites may also reflect variations in richness imposed by the background environments of the sites. Figure 3 compares the observed numbers of taxa at each site (Table 1) with the expected numbers based on modern distribution ranges of the taxa modeled with GIS (geographic information systems) software (see Materials and Methods). Observed richness in the rural sites is nearly identical to the expected richness. In contrast, richness in the urban sites is well below the expected values in most cases, and these differences are significant (*χ^2^* = 15.063, *P* = 0.02). The differences are also significant when the four unclassified sites in the urban group are included (*χ^2^* = 26.466, *P* = 0.003), indicating the urban character of these assemblages.

**Figure 4:**
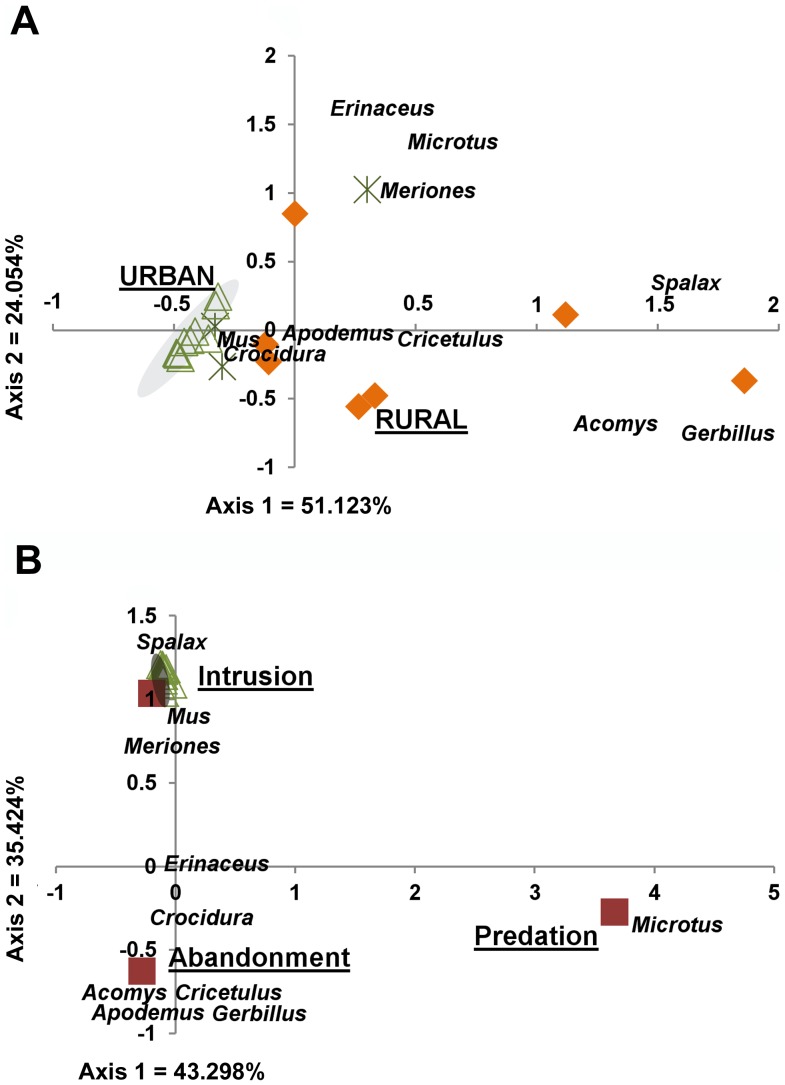
Correspondence analysis of taxonomic composition in assemblages from urban and rural sites. Analysis is based on the frequency data in Table 2. (A) Comparison of assemblages from urban (green triangles), rural (orange diamonds), and unclassified (asterisks) contexts. (B) Comparison of urban and proxy assemblages (purple squares).

To reconstruct taxonomic frequencies we focused on those subsets of the assemblages (Table 2) that are associated most securely with well-defined temporal phases based on ceramic typology, absence of shards diagnostic of earlier or later periods, and archaeological evidence indicating intactness of the deposits. This yields a reduced NISP of 991. Figure 4 A shows the result of correspondence analysis, which reveals a tight cluster combining all of the urban assemblages (Axes1+2 = 75.2% of variance). This cluster is closely associated with occurrences of *Mus* spp. (common mice) and *Crocidura* spp. (white-toothed shrews). All rural sites fall outside the 95% confidence interval ellipse of the urban sites, although there is considerably more variation among them. Among the unclassified assemblages only BU, which represents a deposit in the context of an abandoned urban mound (Table S1), falls within the cluster of urban assemblages based on taxonomic frequencies.

**Table 2:** Taxonomic frequencies based on NISP data, focusing on samples from discrete occupational phases in urban and rural sites and three proxy assemblages.

Settlement type	Site (abb.*)	*Acomys*	*Apodemus*	*Cricetulus*	*Crocidura*	*Erinaceus*	*Gerbillus*	*Meriones*	*Microtus*	*Mus*	*Spalax*	Total
Rural	ERr	18	–	1	4	–	5	13	1	26	17	85
	ERs	12	–	–	–	–	8	4	1	5	7	37
	IS	–	–	–	2	–	–	27	–	45	1	75
	QYb	13	–	–	6	–	6	2	–	46	1	74
	QYc	5	–	2	5	1	1	3	–	64	2	83
	QYd	11	–	–	–	–	2	–	–	34	–	47
Urban	DR1	–	–	–	1	–	–	–	–	18	–	19
	DR2	–	–	–	1	–	–	–	–	24	–	25
	DR3	–	–	–	1	–	–	–	–	21	–	22
	HU	–	–	–	3	–	–	5	1	40	–	49
	KNs	–	–	–	–	–	–	2	–	12	–	14
	KNu	–	–	–	2	–	–	–	–	26	–	28
	MG	–	–	–	4	–	1	3	1	123	3	135
	RHc	–	–	–	1	–	–	1	–	15	–	17
	RHd	–	–	–	4	–	–	2	–	44	–	50
	SH1	–	–	–	5	–	–	–	–	32	–	37
	SH4	–	–	–	1	–	–	1	–	35	–	37
Unclassified	BU	1	–	–	1	–	–	2	1	36	–	41
	HL	–	–	–	–	2	–	8	6	28	6	50
	JR	3	–	–	1	–	–	–	–	37	–	41
	MZ	3	–	–	1	–	–	1	–	19	–	24
Proxy	Pre	2	–	–	7	–	–	1	91	6	–	107
	Int	1	–	–	–	–	–	148	–	63	9	221
	Abn	264	403	5	77	–	224	55	1	86	–	1115

*For site names see Table S1.

An ordination analysis (Figure 4 B) compares the urban assemblages to three proxy assemblages representing different modes of accumulation in abandoned settlements (see Materials and Methods and Taphonomy S1). The urban assemblages are much more closely associated with well-known tunneling taxa such as *Meriones* spp. (jirds) and *Spalax* sp. (mole rats; intrusion assemblage) than with taxa representing communities of abandoned sites or predation assemblages (Axes1+2 = 78.7% of variance). More than revealing statistically significant groupings of sites or taxa, these ordination results indicate quantitative associations among them that are consistent across samples. Thus, *Mus* and *Crocidura* are associated more consistently than other taxa with urban sites; *Meriones* is associated with rural and urban sites (probably abandonment) and with intrusion; *Acomys* sp. (spiny mice) with rural sites (possibly abandonment); and *Microtus* (field voles) with predation and to a lesser extent with rural sites (see also Table 1).

**Figure 5:**
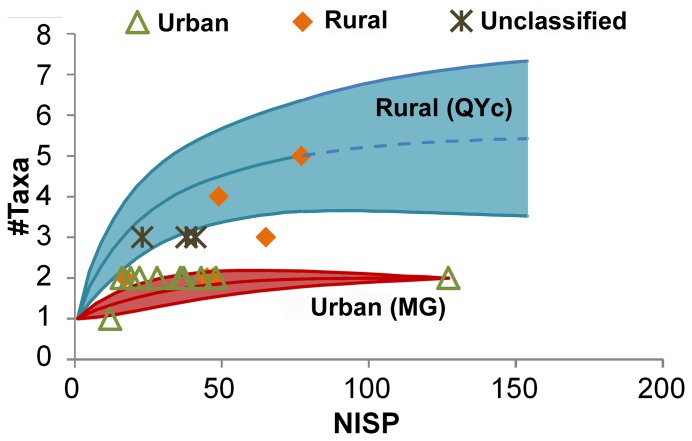
Rarefaction analysis of urban and rural assemblages. Data used for this analysis excludes taxa that are rare in the urban assemblages and indicate abandonment/intrusion (*Meriones*, *Microtus*, *Spalax*, *Gerbillus*). NISP is the number of identified specimens.

We assess taxonomic richness in the urban and rural assemblages by excluding the remains of rare taxa, which are considered likely to be remains from contexts of abandoned urban sites (*Meriones*, *Microtus*, *Spalax*, *Gerbillus* [gerbils]). In Figure 5 we present the data obtained from sample-based rarefaction analysis, which also takes differences in sample size into account. Rarefaction and extrapolation were performed with iNEXT using the Chao1 estimator [34]–[35]. The curve for the urban assemblage with the largest sample size (MG) reaches a clear asymptote, which suggests redundancy in sampling and saturation in taxa. All other urban assemblages have smaller samples, shown only as the end points (green triangles) of their respective curves. These cluster within or near the area enclosed by the confidence intervals of the main urban curve.

The rural curve for the assemblage with the largest sample size (QYc) shows a significantly larger number of taxa than in the urban assemblages across all sample sizes, as indicated by the non-overlapping confidence intervals of the two main curves in Figure 5. With its given sample size the rural curve does not reach the asymptote, and the extrapolated part of the curve (dashed line) predicts a maximum of 5–6 taxa for this assemblage. The other assemblages from rural sites (orange diamonds) show a rather wide distribution of richness values. Three of them (ERs, IS, QYd) fall within or near the area enclosed by the confidence intervals of the urban MG assemblage. The correlation between richness and NISP for the rural assemblages (*r* = 0.729, *P* = 0.1, n = 6) is more apparent than for the urban assemblages (*r* = 0.267, *P* = 0.428, n = 11), but neither is significant. This indicates a stronger sample size effect on richness among the rural than among the urban assemblages. Of the four unclassified cases only one (HL) falls within the cluster of urban assemblages.

To determine whether the archaeological remains of the *Mus* spp. belong to the commensal house mouse (*Mus musculus domesticus*) and not to its sympatric twin, the eastern short-tailed mouse (*Mus macedonicus*), which can display anthropophilous behavior [36], we used a geometric morphometrics technique [37] (see Materials and Methods) to compare the phenotype of 74 M1 teeth from *Mus* remains from different assemblages with those of modern genotyped specimens of *M. m. domesticus* and *M. macedonicus* from Israel. Posterior probabilities >0.95 assigned 95% of the *Mus* material to the *M. m. domesticus* sub-species (Table S2) based on highly significant discrimination of the modern sympatric species (MANOVA, Wilk’s Lambda = 0.087, df1 = 13, df2 = 26, F = 20.78, *P*<0.0001). The rarer *M. macedonicus* was identified in only three assemblages from rural sites (QY, ER) or in an abandonment deposit of an urban site (BU).

## Discussion

Taxonomic richness in archaeozoological assemblages of small mammals from urban sites is significantly lower than in rural sites in the ancient Near East. The distinct *Mus*–*Crocidura* association and the dominance of *Mus* that characterize these urban settlements are accompanied by rare occurrences and low frequencies of other taxa (e.g., *Meriones*, *Acomys*, *Microtus*). The rural sites, on the other hand, all show high frequencies of one or more of these other taxa. This pattern is robust when we consider potential effects of stratigraphic contamination and variation in sample size or background environments of the sites using both taxonomic and taphonomic data. We conclude that the most likely explanation for the observed variation in both taxonomic incidence and frequencies among the study sites is that the ancient urban settlements, during periods of human occupation, were largely inhabited by two species. Geometric morphometric analysis also shows that all of the *Mus* remains from ancient urban sites belong to the commensal house mouse *M. m. domesticus*. The remarkable consistency in occurrence and frequencies of *Mus* and *Crocidura* among urban sites suggests that these two taxa comprised the occupational community of the settlements.

Auffray et al. [36] showed that in Israel, *M. m. domesticus* is associated with settlements whereas the sympatric eastern short-tailed mouse *M. macedonicus* is restricted to agricultural fields and areas less disturbed by humans. Data on the commensalism of *Crocidura* shrews, and especially *C. suaveolens*, are available from urban ecological studies across Europe [38]–[39]. Other taxa occurring in ancient Near Eastern settlements seem to represent various modes of settlement abandonment or rural occupation. *Acomys* occurs in fairly low frequencies in what can be considered as abandonment contexts of urban sites such as silos, and in more substantial frequencies in the rural assemblages, where in some cases it is the second most abundant taxon. This species has been documented in modern settlements in the Near East [40]–[41] and in rural settlement contexts in East Africa [42]–[43], and is probably an indicator of either abandonment or rural settlement occupation. *Meriones* is an abandonment indicator that is also associated with intrusion and stratigraphic contamination. *Microtus* may be linked more distantly with abandonment and intrusion, and shows an especially high frequency only in an assemblage associated with predation. Both *Meriones* and *Microtus* are semi-fossorial taxa that tunnel extensively [44] and typically comprise the most common members of grassland and agricultural environments in the Mediterranean region of Israel, as seen in modern assemblages of barn owl pellets [45].

Comparison of ancient urban and rural settlements suggests that dense and continuous urban settlement over hundreds to thousands of years, and its interaction with the buildup of mounds, depressed species richness relative to rural sites with low-density and discontinuous settlement. Our urban assemblages show remarkable consistency in community structure in spite of the considerable variations in size, geographic location, and time of occupation among these settlements. Even our unclassified assemblages from contexts of abandoned or non-mound urban settings are closely associated with the urban assemblages in some of the analyses. The observed urban pattern of low taxonomic richness may have resulted from the ecological circumstances during periods of settlement occupation, or from the legacy of mound formation due to continuous and dense long-term settlement, or from both.

To provide a historical perspective on changes in the structure of urban ecological communities, we compared ancient and modern data. Data from modern cities reveal a complex pattern, which in some cases involve high species diversity related to the high heterogeneity and patchiness of habitat structure [2]–[5]. This heterogeneity, however, is associated mainly with suburban or urban fringe areas where building density is often low and a sizable proportion of the area is covered by vegetation within habitat patches of varying sizes [7]. Urban core areas, typically characterized by high density of buildings and a high proportion of contiguous impervious surfaces, show low species diversity and domination of commensal species.

The pattern of a small number of abundant commensal species has been documented for urban small mammals in different parts of the world. Dickman [1] showed that in Oxford, England, species richness of small mammals decreased significantly in areas close to buildings and to patches of barren ground. Mahan and O’Connell [46] demonstrated decreasing diversity of small mammals in urban parks along a gradient from least to most urbanized areas in central Pennsylvania, USA. In a study in Buenos Aires, Cavia et al. [47] trapped not more than three species in industrial-residential neighborhoods and in densely settled slums. In each of these areas a single species had a relative abundance of >70–100%. In another Argentinean study across a wide range of habitats in the city of Rio Cuarto, 74% of the total number of captured small mammals were found to belong to only three species [48]. In the cited examples, the abundant species of dense urban environments include widespread cosmopolitan commensals such as the house mouse and rats (*Rattus rattus* or *R. norvegicus*) or indigenous commensals such as *Peromyscus* spp. in the USA.

Our study shows a very similar pattern of low taxonomic richness in ancient urban settlements. Shochat et al. [49] recently argued that urban ecological communities across different taxonomic groups are characterized by the dominance of a few organisms, termed winner species. Their studies of feeding ecology in two North American cities (Phoenix and Baltimore) suggested that ample food supply and probably also the low occurrence of predators within the urban environment removes certain limits on population growth of relatively large and dominant species among birds and spiders. These winner species out-compete many other species, which will consequently undergo reduction in population size and may become excluded altogether from the urban ecosystem [49].

It is unclear how such small-scale ecological mechanisms interact with major drivers of species diversity in urban ecosystems such as the increasing size and density of the human population. The majority of studies in modern urban ecology have focused on describing patterns of variation in the occurrence and abundance of species in relation to variations in land-use types within cities. Other large-scale global studies have examined the relationship between species diversity and human population density used as a correlate of urban intensification [5], [50]. This last approach is confounded, however, by difficulties in distinguishing the impact of urban growth from the effects of background environments.

By looking instead at broad evolutionary trajectories in the ecology of human settlements, we may be able to obtain a fresh perspective on the processes observed in cities today. It is possible that the distinctive pattern of winner species documented by Shochat et al. [49] is not unique to urban settlement, whether ancient or modern. To the best of our knowledge, a distinctly different pattern of species diversity, in which the diversity is greater within settlements than outside them, has been recorded only from small-scale villages of Maasai herders in East Africa [43]. Here the number of people per village is <20 and seasonal abandonment of villages is common. In contrast, small-scale sedentary agricultural villages in Africa show frequencies of >90% for only one or two commensal species including cosmopolitan rats and/or indigenous *Mastomys* sp. [51]–[52]. A similar pattern has already been established in some of the earliest agricultural villages in the Near East and Europe [53]–[54]. Early archaeozoological data from the Near East indicate that the commensal house mouse gradually became the dominant species of small mammal as human societies transitioned from mobile hunting and gathering to sedentary farming [54]. It is possible that the initial transition from mobility to sedentism, with resulting growth in the size and density of human populations, gave rise to the dominance of house mice in areas settled by humans.

Our data indicate that the initial emergence of urbanism probably further enhanced this pattern and reduced species diversity to the rock-bottom levels seen in modern urban core environments. It is known that although the process of mound formation in the Near East began with the appearance of the earliest sedentary settlements, it accelerated dramatically with the rise of urbanization [55]–[56]. This historic transition brought with it profound developments in social organization and technology [11]. Based on our data, we can determine that the distinctive ecology of species-poor communities seen across modern cities was certainly well established in ancient urban settlements in the Near East more than 2,500 years ago.

Our data further point to long-term stability in the structure of urban ecological communities at time-scales of thousands of years. It appears that such communities did not respond to major increases in the size and density of urban settlements or to large-scale technological advancements that reshaped the environments of urban settlements over time. Ecological patterns associated with modern-day low-density suburban development indicate a clear deviation from this persistent pattern. Such low-density sprawl, which incorporates extensive open and vegetated patches into urban fringe environments, increases species diversity in comparison to the urban core [7]. Data on cities during the Middle Ages in England indicate that this pattern of high diversity is not strictly a 20^th^ century phenomenon: Armitage and West [57] uncovered a rich assemblage of the remains of small mammals, including nine different genera, in the gardens of an urban monastery on the outskirts of 15^th^ century London. This finding was related to the properties of urban renewal and growth that characterized England during that period [58], and may represent an early form of urban sprawl.

Better informed decisions regarding conservation and management policies in modern ecosystems should take into account the historical data on the ecology of these environments that are made available through archaeological research [59]–[60]. Fletcher [11] argues that settlement growth in general, and the transition to urbanism in particular, have demonstrated a trajectory of increasing rather than decreasing density at time-scales of thousands of years. We believe that the initial historical shift from low-density agricultural villages to high-density urban settlements was sufficient to reduce species diversity to the rock-bottom levels we see in cities today. The perspective added by the present study with regard to long-term processes in urban ecology may be relevant in the debate over the desirability of modern-day low-density urban sprawl [8]. The surprisingly high levels of species diversity at the fringe of expanding modern cities certainly merit scientific attention. Nonetheless, the data presented here add a significant historical perspective through which to evaluate issues of conservation within cities in view of the negative impacts on species diversity as a result of current urban expansion. The possibility that high diversity in peripheral urban areas arose only recently and represents a short-term phenomenon in urban ecology calls for further research based on long-term data.

## Materials and Methods

The techniques used to document small mammals in ancient settlements differ from those used to monitor living communities in modern settings. This involves the retrieval, taxonomic identification and enumeration of skeletal remains from archaeological sediments. Archaeozoological material also represents time-averaged assemblages less prawn to stochastic effects than modern data which typically represents temporal snapshots. The temporal resolution of archaeological excavation in the Bronze Age or Iron Age in the Near East is typically of a number of decades per the basic vertical unit of excavation. Because ancient urban sites in the Near East are small and homogeneous in comparison to modern cities we focus on the variation among assemblages from different areas of excavation, strata and sites without overarching concern for spatial variation within sites.

A total soil volume close to nine cubic meters was recovered for the purpose of this study. Archaeozoological samples were collected from ongoing excavations by retrieving between one and three buckets of soil from contexts selected for their intact condition based on field observations. This affords partial control regarding potential disturbance due to activities in later periods. Better data on context quality becomes available through post excavation work including careful analysis of field observations and chronologically diagnostic ceramic finds. The soil samples underwent wet-sieving through fine 1 mm mesh screens and laboratory sorting of the residue to collect small skeletal remains. We declare that no permits were required for the described study, which complied with all relevant regulations.

The average number of specimens retrieved from soil samples was 1.75±0.26/10l. This indicates high dispersion of the remains implying that the most appropriate method for quantifying the assemblages is the basic and straightforward number of identified specimens (NISP). In contrast, when archaeozoological assemblages are recovered in large concentrations there is a high likelihood that different skeletal fragments belong to the same skeletal element or individual animal (i.e., redundancy).

The analysis protocol included taxonomic identification to the genus level using the comparative collection of the Laboratory of Archaeozoology, University of Haifa. The rate of taxonomic identification among the assemblages was close to 60% on average (57.21±16.68) using both cranial and post-cranial material. Taphonomic data recorded include skeletal element, complete and burned specimens, presence of chemical corrosion, and aging based on long bone epiphyseal fusion (see Taphonomy S1).

We include in the analysis three proxy assemblages (Table S1) from modern and ancient contexts: 1) Abandonment - data from live-trapping at a long-abandoned settlement site – Khirbet Saadim in the Jerusalem Mts., 2) Intrusion - remains of small mammals that were retrieved from the site of RH and represent species that tunneled into the archaeological layers likely during a period of settlement abandonment, and 3) Predation - remains from an abandoned structure of the Early Bronze Age at the site of MG which were accumulated by a small predator from areas outside of the settlement. The intrusion assemblage consists of unusually high concentrations of the remains of small mammals in three loci where field observations indicate potential disturbances either due to later human excavation activities such as the construction of a wall or the digging of a pit or to a location near the surface of the site. Both the intrusion and predation assemblages were collected through relatively coarse methods which could have involved loss of some of the smaller specimens and potential bias to taxonomic composition. Nonetheless, these assemblages include high abundances of taxa such as *Meriones* or *Microtus* and of complete skeletal specimens which are rare in other archaeozoological assemblages in the study and suggest limited bias related to size. To identify patterned associations among the archaeological and proxy assemblages we employ ordination analysis. Statistical analysis was performed with PAST Paleontological Statistics [61] (Ver. 2.17c) unless indicated otherwise.

Expected numbers of genera per site were derived from present distribution maps of the species, obtained from Mendelssohn and Yom-Tov [62] and based on data collected outside of cities or towns. The maps were converted to GIS layers which were then overlaid, and intersected with the excavation locations in ArcMap software (Ver. 10.1). This procedure enabled us to identify which, and how many, species are expected to be found at each of the sites. These values were compared with the actual number of species found, and a *χ^2^*-test was conducted to assess whether significant differences exist between the observed and expected values.

The taxonomic identification of *Mus* specimens from archaeological *Mus* spp. was performed using a molar shape analysis based on a geometric morphometric approach according to Cucchi et al. [37]. The molar outline of archaeological and modern mice was quantified with a semilandmarks approach [63]. Predictive linear discriminant analyses (LDA) were computed for a referential of the two extant sympatric mouse species of Israel: the house mouse (*M. m. domesticus*) and the eastern short-tailed mouse (*M. macedonicus*). We estimated the classification accuracy of the referential using a leave-one-out cross validation procedure [64] of a canonical variate analysis. Because LDA are affected by sample size and the number of variables used as group predictors [65], we reduced the dimensionality of the variables [66] by defining the number of predictors as the number of first Procrustes coordinates that maximize the variability between groups without overestimating the cross validation percentage as a result of unbalanced sample sizes after 100 repetitions [67]. Specimens classified with posterior probabilities below 0.9 were excluded from the subsequent analyses. Above this threshold, each specimen is assigned to its predicted group in the subsequent analyses.

## Supporting Information

Figure S1 **Correspondence analysis of frequencies of 17 major skeletal elements across 23 assemblages.** Symbols designate: urban (green triangles) rural (orange diamonds), unclassified (asterisks) and proxy (blue squares) assemblages.
(TIF)

Figure S2 **Boxplot of the average proportions of complete specimens from 4 major skeletal elements (humerus, femur, tibia, ulna).** Data is averaged across 23 urban, rural, and proxy assemblages (see data in Table S1).
(TIF)

Table S1 **Study sites: occupational characteristics, context of samples collected for this study, and the state of the archaeozoological remains.**
(DOCX)

Table S2 ***Mus*** spp. molars from the study sites with posterior probabilities^*^ for assignment to ***M. musculus domesticus*** **(DOM) or** ***M. macedonicus*** **(MAC).**
(DOCX)

Taphonomy S1 (DOCX)
