# Supplementary figures and images for: Ancient Urban Ecology Reconstructed from Archaeozoological Remains of Small Mammals in the Near East
Source: PLoS One. 2014 Mar 12;9(3):e91795. doi: 10.1371/journal.pone.0091795 (PMC3951428; doi:10.1371/journal.pone.0091795)

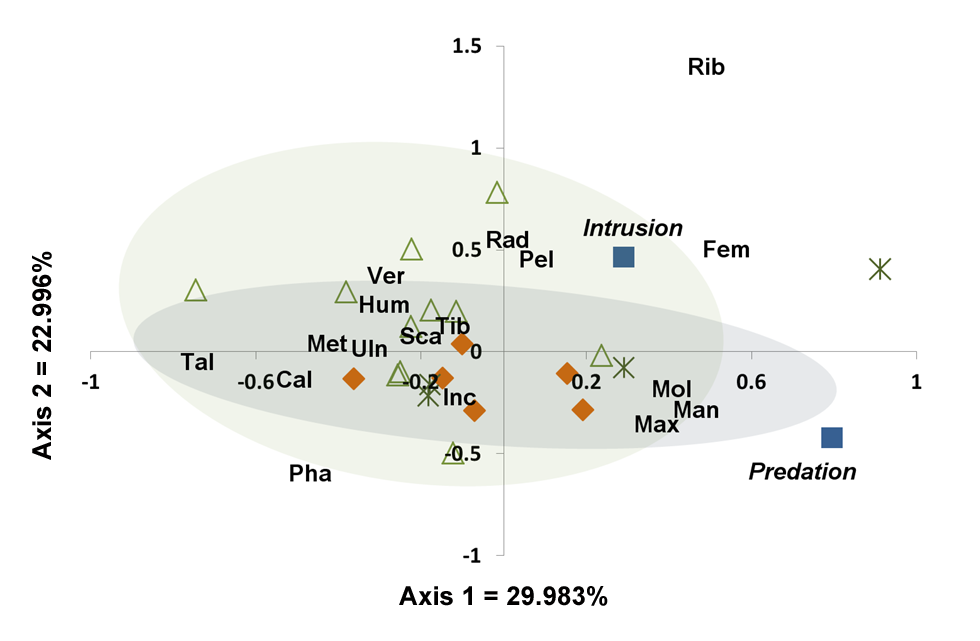

Supplement: Figure S1 — Correspondence analysis of frequencies of 17 major skeletal elements across 23 assemblages. Symbols designate: urban (green triangles) rural (orange diamonds), unclassified (asterisks) and proxy (blue squares) assemblages. (TIF) [file pone.0091795.s001.tif]

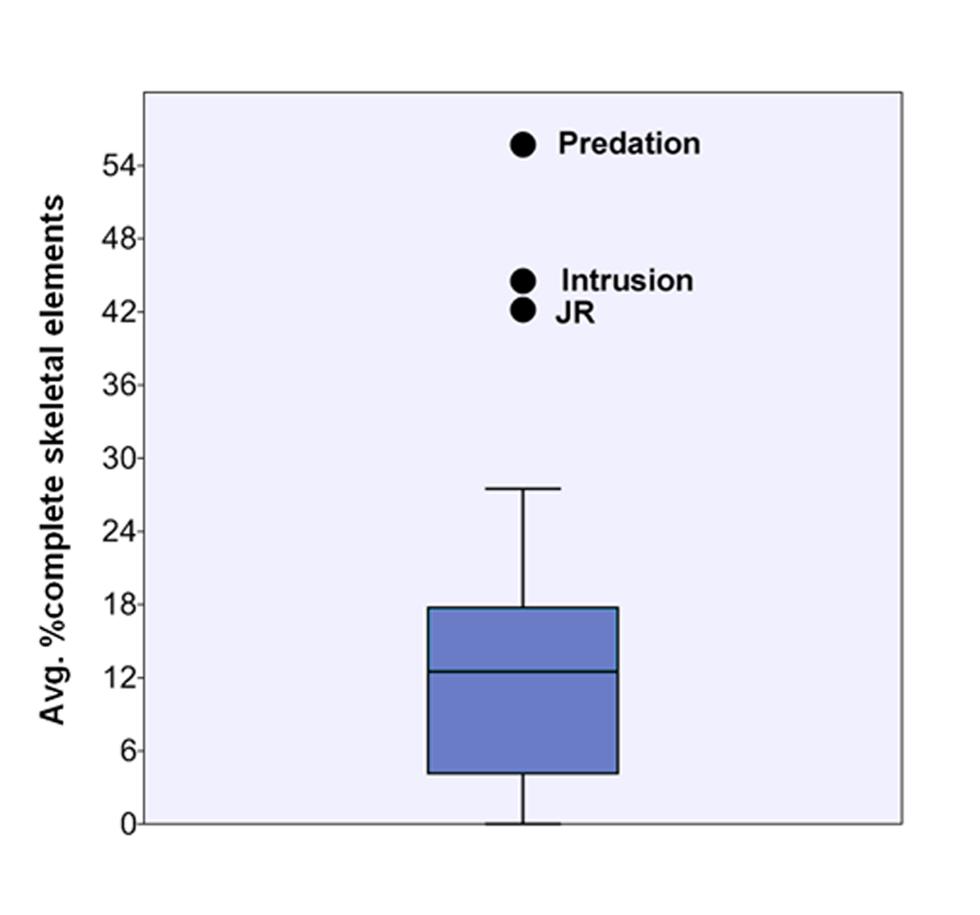

Supplement: Figure S2 — Boxplot of the average proportions of complete specimens from 4 major skeletal elements (humerus, femur, tibia, ulna). Data is averaged across 23 urban, rural, and proxy assemblages (see data in Table S1). (TIF) [file pone.0091795.s002.tif]
