# Supplementary material for: Ancient Urban Ecology Reconstructed from Archaeozoological Remains of Small Mammals in the Near East
Source: PLoS One. 2014 Mar 12;9(3):e91795. doi: 10.1371/journal.pone.0091795 (PMC3951428; doi:10.1371/journal.pone.0091795)
Supplement: Table S1 — Study sites: occupational characteristics, context of samples collected for this study, and the state of the archaeozoological remains. (DOCX) [file pone.0091795.s003.docx]

**Table S1. Study sites: occupational characteristics, context of samples collected for this study, and the state of the archaeozoological remains.**

| **Site name** | **Site abbre-viations** | **Size category*** | **Occupation type** | **Site type** | **Setting** | **Context** | **Excavation unit** | **Excavation area** | **Stratum** | **Period** | **Dates (c. BCE)** | **Additional remarks** | **Avg. %complete** | **No. Burned** | | | **Reference** |
| --- | --- | --- | --- | --- | --- | --- | --- | --- | --- | --- | --- | --- | --- | --- | --- | --- | --- |
|  |  |  |  |  |  |  |  |  |  |  |  |  |  | ***Mus*** | ***Crocidura*** | ***Microtus*** |  |
| Hazor-Lower City | HL | E | Urban | City | Alluvial fan (off-mound) | Residential (off-tel) | Locus | S | 1A | LB IIB | 13th | Settlement extending beyond the mound | 0.00 | 2 | 0 | 1 | [1] |
| Rehov D | RHd | E | Urban | City | Mound | Industrial | Area | D2 | D9a | LB IIB | 13th |  | 4.17 | 2 | 0 | 0 | [2] |
| Dor D12 | DR1 | D | Urban | City | Mound | Residential | Area | D5 | D12 | IA IA early | 12th-11th |  | 0.00 | 0 | 0 | 0 | [3] |
| Dor D11 | DR2 | D | Urban | City | Mound | Residential | Area | D5 | D11 | IA IA late | 12th-11th | Period terminating in site-wide destruction | 16.07 | 13 | 1 | 0 |  |
| Dor D10 | DR3 | D | Urban | City | Mound | Livestock enclosure | Area | D5 | D10 | IA IB | 11th-10th |  | 0.00 | 0 | 0 | 0 |  |
| Kinrot S | KNs | D | Urban | City | Mound | Residential | Area | S | Ve | IA IB | 11th-10th |  | 0.00 | 1 | 0 | 0 | [4] |
| Izbet Sartah | IS | B | Rural | Village | Natural hill | Agricultural installations | Locus | - | II | IA IB/ IA IIA | 11th-9th | Silos may have been filled during abandonment period | 0.00 | 0 | 0 | 0 | [5] |
| Megiddo | MG | D | Urban | City | Mound | Residential | Area | Q | VA-IVB | IA IIA | 10th-9th |  | 17.75 | 5 | 0 | 0 | [6] |
| Rehov C | RHc | E | Urban | City | Mound | Industrial | Area | C | V | IA IIA | 10th-9th | Bee-raising installations | 12.50 | 0 | 0 | 0 | [2] |
| Qeiyafa B | QYb | C | Rural | Fort | Natural hill | Residential & installations | Area | B | IV | IA I/ IA IIA | 11th-10th | A short-lived military outpost | 8.57 | 0 | 0 | 0 | [7] |
| Qeiyafa C | QYc | C | Rural | Fort | Natural hill | Residential & installations | Area | C | IV | IAI/ IA IIA | 11th-10th |  | 12.78 | 0 | 0 | 0 |  |
| Qeiyafa D | QYd | C | Rural | Fort | Natural hill | Residential & installations | Area | D | IV | IA I/ IA IIA | 11th-10th |  | 15.63 | 0 | 0 | 0 |  |
| Motza | MZ | C | Urban-rural | Adminstra-tive center | Hill side | Agricultural installations | Locus | D | V | IA IIB | 8th-6th |  | 25.00 | 0 | 0 | 0 | [8] |
| Hazor-Upper City | HU | D | Urban | City | Mound | Residential | Locus | M | Va | IA III | 8th |  | 5.00 | 0 | 0 | 0 | [9] |
| Burna | BU | C | Urban | Town | Mound | Agricultural installations | Locus | A | - | IA IIB | 8th | Silo may have been filled during abandonment | 0.00 | 0 | 0 | 0 | [10] |
| Er-Ras | ER | B | Rural | Village | Hill side | Residential & installations | Area | F, G | - | Hel | 2nd-3rd | Farmstead | 13.81 | 0 | 1 | 0 | [11] |
| Shiqmona I | SH1 | B | Urban | Town | Mound | Residential | Area | B | I | IA II | 10th-6th |  | 27.50 | 0 | 0 | 0 |  |
| Kinrot U | KNu | D | Urban | Town | Mound | Residential | Area | U | Vt | IA IB | 10th |  | 12.50 | 1 | 0 | 0 | [4] |
| Jerusalem | JR | D | Urban | City | Mound | Vacant margins of city | Locus | Hewn pool | - | IA II late | 9th | Location adjacent to city fortifications | 42.19 | 0 | 0 | 0 | [13] |
| Predation | Pre | D | Urban | City | Mound | Abandoned administrative area | Area | J | 4-5 | EB IB | Early 3rd millenium | Remains accumulated by a predator inhabiting an abandoned structure | 55.72 | 0 | 0 | 0 | [14] |
| Intrusion | Int | E | Urban | City | Mound | Residential & industrial | Site | A, B, C, D, E, F, G, Z | - | LB-IA | 13th-6th | Spatially dispersed sampling using 4-mm sieves | 44.53 | 0 | 0 | 0 | [15] |
| Abandon-ment | Abn | B | Rural | Village | Natural hill | - | - | - | - | Middle Ages | 1st millenium AD | Live-trapping data from an archaeological site | - | - | - | - | [16] |

*Site size categories are ordered B-E (from small to large).

**References:**

1. Zuckerman S (2013) Area S: Renewed excavations in the lower city of Hazor. Near East Archaeol 76: 94–97.
2. Mazar A, Bruins HJ, Panitz-Cohen N, Van Der Plicht J (2005) Ladder of time at Tel Rehov: Stratigraphy, archaeological context, pottery and radiocarbon dates. In: Levy TE, Higham T, editors. The Bible and Radiocarbon Dating: Archaeology, Text and Science. London: Equinox. pp. 193–255.
3. Gilboa A, Sharon I (2008) Between the Carmel and the sea: Tel Dor’s Iron Age reconsidered. Near East Archaeol 71: 146–170.
4. Münger S, Zangenberg, J, Pakkala J (2011) Kinneret—an urban center at the crossroads: Excavations on Iron IB Tel Kinrot at the Lake of Galilee. Near East Archaeol 74: 68–90.
5. Finkelstein I (1986) “Izbet Sartah: An Early Iron Age Site Near Rosh Ha”ayin, Israel. Oxford: British Archaeological Reports.
6. Finkelstein I, Ussishkin D, Cline E (2013) Megiddo V: The 2004-2008 Seasons. Tel Aviv: Emery and Claire Yass Publications in Archaeology.
7. Garfinkel Y, Streit K, Ganor S, Hasel MG (2012) State formation in Judah: Biblical tradition, modern historical theories, and radiometric dates at Khirbet Qeiyafa. Radiocarbon 54: 359–369.
8. Greenhut Z, De-Groot A, Barzilay E (2009) Salvage Excavations at Tel Moẓa: The Bronze and Iron Age Settlements and Later Occupations. Jerusalem: Israel Antiquities Authority.
9. Ben-Tor A (2013) The renewed Hazor excavations. Near East Archaeol 76: 66–67.
10. Shai I, Cassuto D, Dagan A, Uziel, Joe (2012) The fortifications at Tel Burna: Date, function and meaning. Isr Explor J 62: 141–157.
11. Edelstein G, Milevski I (1994) The rural settlement of Jerusalem re-evaluated: Surveys and excavations in the Reph’aim Valley and Mevasseret Yerushalayim. Palest Explor Q 126: 2–23.
12. Elgavish J (1994) Shiqmona on the Seacoast of Mount Carmel. Tel Aviv.
13. Reich R, Shukron E, Lernau O (2007) Recent discoveries in the City of David, Jerusalem. Isr Explor J 57: 153–169.
14. Weissbrod L (2013) The micromammalian remains. In: Finkelstein I, Ussishkin D, Cline EH, editors. Megiddo V: the 2004-2008 Seasons, Volume III. Tel Aviv: Emery and Claire Yass Publications in Archaeology. pp. 1210–1214.
15. Weissbrod L (2014) Microvertebrate assemblages from the Late Bronze and Iron Ages. In: Mazar A, editor. Tel Rehov, Volume 1. Jerusalem: Israel Exploration Society. In press.
16. Ritte U (1964) The dynamics of a population of small mammals in the Judean Hills. Unpublished master's thesis, The Hebrew University of Jerusalem.
