# Supplementary material for: Ancient Urban Ecology Reconstructed from Archaeozoological Remains of Small Mammals in the Near East
Source: PLoS One. 2014 Mar 12;9(3):e91795. doi: 10.1371/journal.pone.0091795 (PMC3951428; doi:10.1371/journal.pone.0091795)
Supplement: Table S2 — Mus spp. molars from the study sites with posterior probabilities* for assignment to M. musculus domesticus (DOM) or M. macedonicus (MAC). (DOCX) [file pone.0091795.s004.docx]

**Table S2. *Mus* spp. molars from the study sites with posterior probabilities^*^ for assignment to *M. musculus domesticus* (DOM) or *M. macedonicus* (MAC).**

|  | 6PCs | | | | | 10PCs | | | | | 13PCs | | | | |  |
| --- | --- | --- | --- | --- | --- | --- | --- | --- | --- | --- | --- | --- | --- | --- | --- | --- |
| Specimen ID |  | | DOM | | MAC |  | | DOM | | MAC |  | | DOM | | MAC |  |
| BU_#10529 | DOM | | 1 | | 0 | DOM | | 1 | | 0 | DOM | | 1 | | 0 |  |
| BU_#10615 | DOM | | 0.98 | | 0.02 | MAC | | 0.01 | | 0.99 | MAC | | 0.01 | | 0.99 |  |
| DR_#2255 | DOM | | 1 | | 0 | DOM | | 1 | | 0 | DOM | | 1 | | 0 |  |
| DR_#2303 | DOM | | 1 | | 0 | DOM | | 1 | | 0 | DOM | | 1 | | 0 |  |
| DR_#2304 | DOM | | 1 | | 0 | DOM | | 1 | | 0 | DOM | | 1 | | 0 |  |
| DR_#2443 | DOM | | 1 | | 0 | DOM | | 1 | | 0 | DOM | | 1 | | 0 |  |
| DR_#2444 | DOM | | 1 | | 0 | DOM | | 1 | | 0 | DOM | | 1 | | 0 |  |
| DR_#2445 | DOM | | 1 | | 0 | DOM | | 1 | | 0 | DOM | | 1 | | 0 |  |
| DR_#2446 | DOM | | 1 | | 0 | DOM | | 1 | | 0 | DOM | | 1 | | 0 |  |
| DR_#4070 | DOM | | 1 | | 0 | DOM | | 1 | | 0 | DOM | | 1 | | 0 |  |
| DR_#4105 | DOM | | 1 | | 0 | DOM | | 1 | | 0 | DOM | | 1 | | 0 |  |
| DR_#4112 | DOM | | 1 | | 0 | DOM | | 1 | | 0 | DOM | | 1 | | 0 |  |
| DR_#4118 | DOM | | 1 | | 0 | DOM | | 1 | | 0 | DOM | | 1 | | 0 |  |
| DR_#4119 | DOM | | 0.99 | | 0.01 | DOM | | 1 | | 0 | DOM | | 1 | | 0 |  |
| DR_#4120 | DOM | | 1 | | 0 | DOM | | 1 | | 0 | DOM | | 1 | | 0 |  |
| DR_#4122 | DOM | | 1 | | 0 | DOM | | 1 | | 0 | DOM | | 1 | | 0 |  |
| DR_#4125 | DOM | | 1 | | 0 | DOM | | 1 | | 0 | DOM | | 1 | | 0 |  |
| DR_#4129 | DOM | | 1 | | 0 | DOM | | 1 | | 0 | DOM | | 1 | | 0 |  |
| DR_#4142 | DOM | | 1 | | 0 | DOM | | 1 | | 0 | DOM | | 1 | | 0 |  |
| DR_#4155 | DOM | | 0.99 | | 0.01 | DOM | | 1 | | 0 | DOM | | 1 | | 0 |  |
| DR_#4158 | DOM | | 1 | | 0 | DOM | | 1 | | 0 | DOM | | 1 | | 0 |  |
| DR_#4160 | DOM | | 1 | | 0 | DOM | | 1 | | 0 | DOM | | 1 | | 0 |  |
| DR_#4166 | DOM | | 0.98 | | 0.02 | DOM | | 1 | | 0 | DOM | | 1 | | 0 |  |
| HL_#9787 | DOM | | 1 | | 0 | DOM | | 1 | | 0 | DOM | | 1 | | 0 |  |
| HU_#1237 | DOM | | 1 | | 0 | DOM | | 1 | | 0 | DOM | | 1 | | 0 |  |
| HU_#1272 | DOM | | 1 | | 0 | DOM | | 1 | | 0 | DOM | | 1 | | 0 |  |
| HU_#969 | DOM | | 1 | | 0 | DOM | | 1 | | 0 | DOM | | 1 | | 0 |  |
| IS_#1160 | DOM | | 1 | | 0 | DOM | | 1 | | 0 | DOM | | 1 | | 0 |  |
| IS_#1643 | DOM | | 1 | | 0 | DOM | | 1 | | 0 | DOM | | 1 | | 0 |  |
| IS_#1645 | DOM | | 1 | | 0 | DOM | | 1 | | 0 | DOM | | 1 | | 0 |  |
| IS_#1688 | DOM | | 1 | | 0 | DOM | | 1 | | 0 | DOM | | 1 | | 0 |  |
| KN_#10215 | DOM | | 0.96 | | 0.04 | DOM | | 1 | | 0 | DOM | | 1 | | 0 |  |
| KN_#10268 | DOM | | 1 | | 0 | DOM | | 0.88 | | 0.12 | DOM | | 0.88 | | 0.12 |  |
| KN_#10315 | DOM | | 0.99 | | 0.01 | DOM | | 1 | | 0 | DOM | | 1 | | 0 |  |
| KN_#10319 | DOM | | 0.98 | | 0.02 | DOM | | 1 | | 0 | DOM | | 1 | | 0 |  |
| KN_#10403 | DOM | | 1 | | 0 | DOM | | 1 | | 0 | DOM | | 1 | | 0 |  |
| KN_#10442 | DOM | | 1 | | 0 | DOM | | 1 | | 0 | DOM | | 1 | | 0 |  |
| KN_#10457 | DOM | | 1 | | 0 | DOM | | 1 | | 0 | DOM | | 1 | | 0 |  |
| KN_#10468 | DOM | | 1 | | 0 | DOM | | 1 | | 0 | DOM | | 1 | | 0 |  |
| MG_#2695 | DOM | | 1 | | 0 | DOM | | 1 | | 0 | DOM | | 1 | | 0 |  |
| MG_#2697 | DOM | | 1 | | 0 | DOM | | 1 | | 0 | DOM | | 1 | | 0 |  |
| MG_#2782 | DOM | | 1 | | 0 | DOM | | 1 | | 0 | DOM | | 1 | | 0 |  |
| MG_#2783 | DOM | | 0.99 | | 0.01 | DOM | | 1 | | 0 | DOM | | 1 | | 0 |  |
| MG_#3004 | DOM | | 1 | | 0 | DOM | | 1 | | 0 | DOM | | 1 | | 0 |  |
| MG_#3007 | DOM | 1 | | 0 | | | DOM | 1 | 0 | | | DOM | | 1 | 0 | |
| MG_#3083 | DOM | 1 | | 0 | | | DOM | 1 | 0 | | | DOM | | 1 | 0 | |
| MG_#3175 | DOM | 1 | | 0 | | | DOM | 1 | 0 | | | DOM | | 1 | 0 | |
| MG_#3296 | DOM | 1 | | 0 | | | DOM | 1 | 0 | | | DOM | | 1 | 0 | |
| MG_#3590 | DOM | 1 | | 0 | | | DOM | 1 | 0 | | | DOM | | 1 | 0 | |
| MG_#3636 | DOM | 1 | | 0 | | | DOM | 1 | 0 | | | DOM | | 1 | 0 | |
| MG_#3792 | DOM | 1 | | 0 | | | DOM | 1 | 0 | | | DOM | | 1 | 0 | |
| MZ_#10123 | DOM | 1 | | 0 | | | DOM | 1 | 0 | | | DOM | | 1 | 0 | |
| MZ_#10135 | DOM | 1 | | 0 | | | DOM | 1 | 0 | | | DOM | | 1 | 0 | |
| QY_#373 | DOM | 1 | | 0 | | | DOM | 1 | 0 | | | DOM | | 1 | 0 | |
| QY_#374 | DOM | 1 | | 0 | | | DOM | 1 | 0 | | | DOM | | 1 | 0 | |
| QY_#527 | DOM | 1 | | 0 | | | DOM | 1 | 0 | | | DOM | | 1 | 0 | |
| QY_#543 | DOM | 1 | | 0 | | | DOM | 1 | 0 | | | DOM | | 1 | 0 | |
| QY_#641 | DOM | 0.99 | | 0.01 | | | DOM | 1 | 0 | | | DOM | | 1 | 0 | |
| QY_#645 | DOM | 1 | | 0 | | | DOM | 1 | 0 | | | DOM | | 1 | 0 | |
| QY_#743 | DOM | 1 | | 0 | | | DOM | 1 | 0 | | | DOM | | 1 | 0 | |
| QY_#89 | DOM | 1 | | 0 | | | DOM | 1 | 0 | | | DOM | | 1 | 0 | |
| QY_#946 | MAC | 0 | | 1 | | | MAC | 0 | 1 | | | MAC | | 0 | 1 | |
| ER_#13408 | DOM | 1 | | 0 | | | DOM | 1 | 0 | | | DOM | | 1 | 0 | |
| ER_#13587 | DOM | 1 | | 0 | | | DOM | 0.95 | 0.05 | | | DOM | | 0.95 | 0.05 | |
| ER_#14041 | MAC | 0 | | 1 | | | MAC | 0 | 1 | | | MAC | | 0 | 1 | |
| RH_Int | DOM | 1 | | 0 | | | DOM | 1 | 0 | | | DOM | | 1 | 0 | |
| RH_#1721 | DOM | 0.99 | | 0.01 | | | DOM | 1 | 0 | | | DOM | | 1 | 0 | |
| RH_#1786 | DOM | 1 | | 0 | | | DOM | 1 | 0 | | | DOM | | 1 | 0 | |
| RH_#1833 | DOM | 0.99 | | 0.01 | | | DOM | 1 | 0 | | | DOM | | 1 | 0 | |
| RH_#1970 | DOM | 1 | | 0 | | | DOM | 1 | 0 | | | DOM | | 1 | 0 | |
| RH_#2037 | DOM | 0.99 | | 0.01 | | | DOM | 1 | 0 | | | DOM | | 1 | 0 | |
| SH_#11374 | DOM | 1 | | 0 | | | DOM | 1 | 0 | | | DOM | | 1 | 0 | |
| SH_#11841 | DOM | 0.99 | | 0.01 | | | DOM | 1 | 0 | | | DOM | | 1 | 0 | |
| SH_#12768 | DOM | 1 | | 0 | | | DOM | 1 | 0 | | | DOM | | 1 | 0 | |

^*^Assignment to *M. musculus domesticus* (DOM) or *M. macedonicus* (MAC) is based on discriminant function analysis performed on a dataset of molar shape variables with 6, 10, and 13 principal components, which provide 100% correct classification using a cross-validation approach. These components were used together to check the stability of the assignments. Specimen numbers classified as MAC are underlined.
