# Supplementary material for: Ancient Urban Ecology Reconstructed from Archaeozoological Remains of Small Mammals in the Near East
Source: PLoS One. 2014 Mar 12;9(3):e91795. doi: 10.1371/journal.pone.0091795 (PMC3951428; doi:10.1371/journal.pone.0091795)
Supplement: Taphonomy S1 — (DOCX) [file pone.0091795.s005.docx]

**Taphonomy S1**

We see considerable uniformity among the urban and rural sites in the occurrence of different skeletal elements and levels of fragmentation suggesting a generalized occupational taphonomic mode. A correspondence analysis of skeletal element frequencies based on counts of specimens shows that the variation among the rural assemblages is nearly engulfed by the variation among the urban assemblages (Axes1+2 = 52.9% of variance; Figure S1). Only two assemblages fall outside the 95% confidence interval ellipses: the JR assemblage from a peripheral vacant part of the settlement and the archaeological proxy assemblage representing predation. The association of these two assemblages with occurrences of the upper and lower jaws and the relatively low occurrence of jaws in the urban and rural assemblages may indicate the impact of trampling on the latter occupational groups [1]. Similarly, the JR assemblage also contains an especially high average proportion of complete or nearly complete specimens from four major skeletal elements (mandible, maxilla, femur, and humerus; Table S1 and Figure S2). Completeness is also high in the two archaeological proxy assemblages representing predation and intrusion, which are associated with abandonment.

Burned remains which indicate an association with human activities are generally uncommon among the assemblages (NISP = 28) and except for a single specimen of *Microtus* all of them belong to *Mus* and *Crocidura*, the two most ubiquitous taxa among the occupational assemblages (Supplementary Table 1). It may also be important that only one of the burned specimens occurs at a rural site whereas the rest are distributed among different urban sites. Moreover, a concentration of burned remains is observed in association with a burned down structure at the site of DR where extensive parts of the deposits containing the remains were sealed under the destruction debris.

We did not detect the diagnostic digestion marks associated with predators such as nocturnal or diurnal raptors and small mammalian carnivores [1]. Some form of corrosion of skeletal remains which may indicate the impact of digestion by a predator or other post-depositional corrosive forces is uncommon occurring among 55 specimens in both urban and rural sites. In addition, aging based on epiphysial fusion shows that both early and late fusing skeletal parts (early: distal femur; late: proximal ulna, humerus, and tibia) have close to twice as many unfused specimens as fused ones, a ratio which is consistent across different urban and rural sites. In contrast, the late fusing proximal femur has roughly equivalent numbers of fused and unfused specimens in the different assemblages. This finding indicates possible improvement in survivorship among older juveniles which among house mice may be considered an indicator of commensal populations [2].

**References:**

1. Andrews P (1990) Owls, Caves, and Fossils: Predation, Preservation, and Accumulation of Small Mammal Bones in Caves. Chicago: University of Chicago Press.
2. Courtney PA, Fenton MB (1976) The effects of a small rural garbage dump on populations of *Peromyscus Leucopus* Rafinesque and other small mammals. J Appl Biol 13: 413–422.
